# The Use of Skype Video Telecommunication (VTC) for Social Visits in a Medium Secure Hospital: A Service Evaluation

**DOI:** 10.1192/bjo.2022.410

**Published:** 2022-06-20

**Authors:** Obinna Okonkwo, Simon Gibbon, Lucy McCarthy, Nicholas Taylor

**Affiliations:** Arnold Lodge, Leicester, United Kingdom

## Abstract

**Aims:**

The COVID-19 pandemic brought unprecedented disruptions in the ways we lived and interacted with one another. Research studies done in the immediate aftermath suggested that the COVID-19 pandemic and associated lockdown restrictions may have increased feelings of isolation and loneliness, which together with disruptions in services may have precipitated psychological distress and mental health deterioration, particularly among persons with pre-existing mental health conditions. Following the introduction of first national lockdown in late March 2020, all visits to the hospital by family and friends were ceased. VTC became one of the rapid interventions implemented across several NHS Hospitals to promote continued patient contact with carers. In October 2021, we set out to undertake an evaluation project to determine the level of patient satisfaction with the use of Skype for social visits, to understand patient and staff perspectives on its pros and cons, and to understand patient preference post-COVID-19 pandemic.

**Methods:**

All ward-based staff who had ever facilitated Skype social visits and all patients who had had at least one social visit facilitated by Skype were approached to participate in the project. Data were collected using anonymous questionnaires with both quantitative and qualitative items.

**Results:**

A total of twenty-nine patients and thirty-nine nursing staff participated in the study.

Sixty-two per cent of patient-participants reported being satisfied with the Skype social visits and over half (52%) rated the Skype social visits as ‘the same’ as face-to-face visits. All participants reported patient-satisfaction with the process and speed of setting up a Skype visit, the benefits of visual contact and the reduction of travel costs. A few patient-participants noted that they relished the opportunity of seeing their home environment. Issues regarding increased demands on staffing resources, privacy, IT skills, and hardware and software glitches were identified.

Overall, Skype social visits have been a positive experience for the patients and have not resulted in any significant risk concerns. Most patients (90%) indicated that they would like Skype social visits to continue post-COVID-19 pandemic.

**Conclusion:**

The average length of stay (LOS) of patients is often longer in forensic compared to general adult mental health units and about 4.5 years at the study site. This evaluation found that the introduction of Skype for the purposes of social visits was considered a useful development by both patients and staff. The study findings were fed back to all stakeholders and certain changes have been implemented as a result.

